# Caveolin-1 identified as a key mediator of acute lung injury using bioinformatics and functional research

**DOI:** 10.1038/s41419-022-05134-8

**Published:** 2022-08-06

**Authors:** Lihua Qu, Yi Li, Chao Chen, Tong Yin, Qian Fang, Yijin Zhao, Wenting Lv, Ziqi Liu, Yangye Chen, Li Shen

**Affiliations:** 1 Department of Physiology, Hunan Normal University School of Medicine, Changsha, 410013 China; 2 Department of Pathogenic Biology, School of Basic Medical Sciences, Wuhan University, Wuhan, 430071 China; 3 School of Medicine & Holistic Integrative Medicine, Nanjing University of Chinese Medicine, Nanjing, 210013 China

**Keywords:** Cell signalling, Respiratory tract diseases

## Abstract

Acute lung injury (ALI) is a potentially life-threatening, devastating disease with an extremely high rate of mortality. The underlying mechanism of ALI is currently unclear. In this study, we aimed to confirm the hub genes associated with ALI and explore their functions and molecular mechanisms using bioinformatics methods. Five microarray datasets available in GEO were used to perform Robust Rank Aggregation (RRA) to identify differentially expressed genes (DEGs) and the key genes were identified via the protein-protein interaction (PPI) network. Lipopolysaccharide intraperitoneal injection was administered to establish an ALI model. Overall, 40 robust DEGs, which are mainly involved in the inflammatory response, protein catabolic process, and NF-κB signaling pathway were identified. Among these DEGs, we identified two genes associated with ALI, of which the CAV-1/NF-κB axis was significantly upregulated in ALI, and was identified as one of the most effective targets for ALI prevention. Subsequently, the expression of CAV-1 was knocked down using AAV-shCAV-1 or CAV-1-siRNA to study its effect on the pathogenesis of ALI in vivo and in vitro. The results of this study indicated that CAV-1/NF-κB axis levels were elevated in vivo and in vitro, accompanied by an increase in lung inflammation and autophagy. The knockdown of CAV-1 may improve ALI. Mechanistically, inflammation was reduced mainly by decreasing the expression levels of CD3 and F4/80, and activating autophagy by inhibiting AKT/mTOR and promoting the AMPK signaling pathway. Taken together, this study provides crucial evidence that CAV-1 knockdown inhibits the occurrence of ALI, suggesting that the CAV-1/NF-κB axis may be a promising therapeutic target for ALI treatment.

## Introduction

Acute Lung Injury (ALI) is a serious respiratory illness that is commonly secondary to the systemic inflammatory response [1, 2]. The causes of ALI include infection, trauma, aspiration, and transfusion [3]. ALI can lead to severe acute respiratory distress syndrome (ARDS). Abrupt onset, quick development, and poor prognosis have caused the ARDS death to be as high as 30–40% [4]. However, the pathology of ALI is complicated and has not been fully elucidated. There is no specific method of treatment for ALI [5]. Common therapies mainly include primary disease management, mechanical ventilation, administration of vasodilators, surfactants, antioxidants, glucocorticoids, and anti-inflammatory drugs [6]. However, due to the high death rate, these traditional therapies are not sufficient [7, 8]. Improving our understanding of the mechanisms involved in ALI and the identification of pathological modulators are crucial for the studies of ALI [9, 10]. Therefore, analysis via bioinformatics may help explore the potential mechanisms involved in ALI and help identify novel targets for use in future clinical research.

Caveolin-1 (CAV-1) is the primary constituent of caveolae and plays a crucial role in maintaining its shape, structure, and function [11, 12]. Studies have found that CAV-1 is involved in multiple processes, including transmembrane transport, endocytosis, lipid metabolism, and signal transduction [13–15]. Importantly, CAV-1 functions as a mediator of many signaling pathways that lead to the activation of nuclear factor kappa B (NF-κB) [16]. NF-κB is a multi-protein complex and NF-κBp65 (RELA) is one of its most important constituents. As a primary transcription factor, NF-κBp65 participates in the regulation of various inflammatory mediators [17–19], while inflammation plays an important role in the development of ALI [20, 21]. Garrean et al. [22] found that the knockdown of CAV-1 suppresses the activation of NF-κB and inhibits the infiltration of inflammatory cells, to reduce overall mortality. Meanwhile, it has also been reported that the promotion of CAV-1 expression suppresses MAPK and NF-κB activation, alleviating pulmonary inflammation [23]. However, the exact mechanism by which the CAV-1/NF-κB axis is involved in ALI is still not clear.

**Fig. 1:**
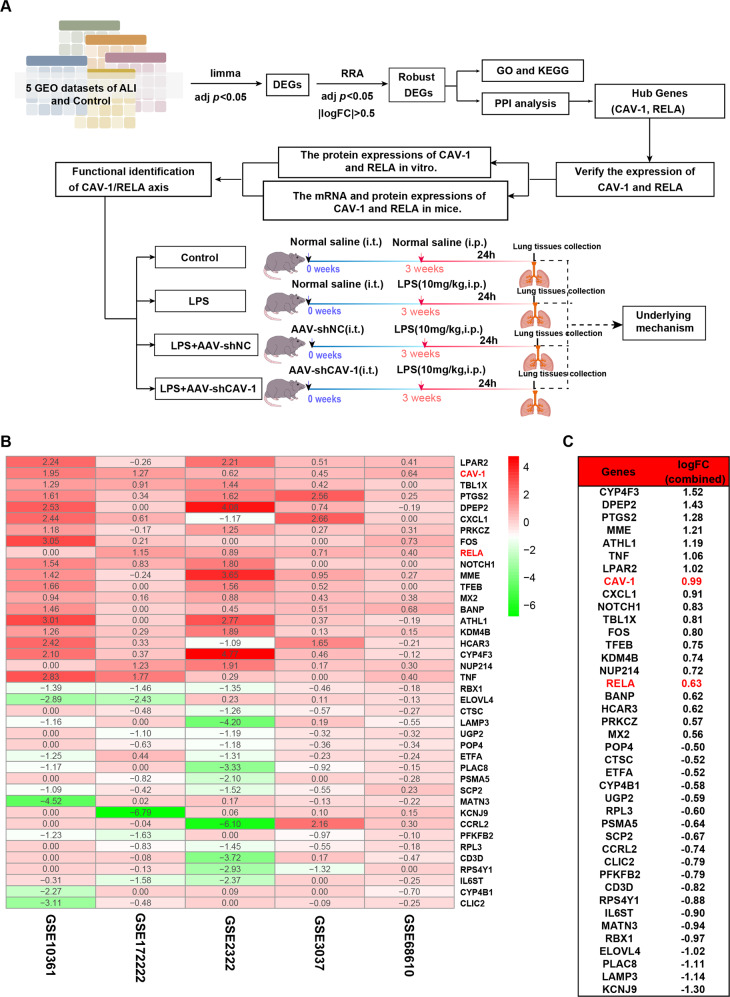
The research scheme and RRA analysis of DEGs in ALI. **A** Research scheme and a flow framework. We analyzed 5 ALI datasets using bioinformatics, screened out common differential genes, and performed GO, KEGG, and PPI analysis on these differential genes. The mRNA and protein expressions of the CAV-1 and RELA were verified in vitro and in vivo. The functions of CAV-1 and RELA were further verified to explore the potential mechanism in ALI. **B** Heatmap demonstrated the 40 robust DEGs between the ALI and control. Twenty genes were promoted and twenty genes were suppressed in ALI. Red shows upregulated gene expression and green shows downregulated gene expression in ALI. Numbers in the heatmap indicate log2 FC of genes in the five datasets compared with control groups. **C** The log2 FC of 40 DEGs was calculated from five datasets. The log2 FC sums of CAV-1 and RELA were 0.99 and 0.63, suggesting these two genes were significantly upregulated in ALI.

In this study, we used bioinformatics methods to analyze online clinical five datasets and found that the differential expression of CAV-1 and NF-κBp65 was important for ALI. Furthermore, we further validated the roles of CAV-1 and NF-κBp65 in vivo and in vitro, while we also explored the potential mechanisms by which CAV-1 could provide novel targets for ALI treatment (Fig. 1A).

## Materials and methods

### GEO datasets retrieval and DEG identification

Microarray data were obtained from the Gene Expression Omnibus (GEO) database (https://www.ncbi.nlm.nih.gov/geo/). Five datasets on the gene expression of ALI patients and healthy controls, including GSE68610 [24], GSE3037 [25], GSE172222 [26], GSE2322 [27], and GSE10361 [https://www.ncbi.nlm.nih.gov/geo/query/acc.cgi] were obtained.

All data in the microarray datasets were processed using R language and the fold change between lung injury patients and controls were calculated. Identification of differentially expressed genes was performed using the R package, “limma”, and a *p* value < 0.05 was used to identify the differentially expressed genes (DEGs). Robust Rank Aggregation (RRA) was used to further identify robust DEGs, and a *p* value of <0.05 and log|FC| > 0.5 were considered to indicate significance.

### Functional enrichment analysis of the DEGs

Gene ontology (GO) and Kyoto encyclopedia of genes and genomes (KEGG) enrichment analysis of DEGs were performed using the R package, “Clusterprofiler”. A *p* value < 0.05 was considered to indicate statistical significance.

### Construction of the PPI network

The tool for the retrieval of interacting genes (STRING, v11.5) is a database that can be used to search for and visualize networks of proteins and was used in this study to perform the protein-protein interaction (PPI) analysis. The networks were further visualized using the Cytoscape plug-in, “Cytohubba”, in which the degree of each node was illustrated using specific colors.

### Construction of animal models and experimental procedures

Male 7–8 week old C57BL/6J mice were obtained from Hunan SJA Laboratory Animal CO., Ltd (Changsha, China). This study has been approved by the Ethics Committee for Animal Experiments of Hunan Normal University. 40 mice were randomly separated into four groups based on their weight. The four groups were the control, lipopolysaccharide (LPS), LPS + AAV-shNC, and LPS + AAV-shCAV-1 groups. The mice were treated with adeno-associated virus 6 through intratracheal instillation for 3 weeks, and the control group was treated with an equal amount of saline. Mice from each group received an intraperitoneal injection of LPS (10 mg/kg) and were killed 24 h later.

### Lung edema

The level of lung edema in the mice was quantified by calculating the lung weight coefficient. After the mice were executed, whole lung tissues were immediately collected and weighed using an electronic balance. The lung weight coefficient was calculated as the wet weight of lung/the weight of the mouse × 100%.

### Enzyme-linked immunosorbent assay (ELISA)

The expression levels of the cytokines, TNF-α, IL-1β, IL-6, and IL-18 were evaluated by ELISA following the manufacturer’s instructions (Beyotime, Shanghai, China). The evaluation was performed on 4 duplicates of each group to ensure constancy.

### Haematoxylin-eosin (H&E) staining and histological assessment

Lung tissues of the mice were fixed using 4% formalin and embedded in paraffin followed by sectioning into 4-μm sections. The tissues were stained using H&E following standard procedures. The pathological characteristics of the tissues were evaluated by light microscopy. The degree of injury of lung tissue was divided into grades 0–4 [28]. Grade 0: no injury; grade 1: injury in <25% of the field of observation; grade 2: injury in 25–50% of the field of observation; grade 3: injury in 50–75% of the field of observation; grade 4: injury throughout the field of observation. At least five fields of observation were randomly selected and analyzed in each sample.

### Immunohistochemistry analysis

Lung tissues were obtained from the mice and embedded in paraffin. After dewaxing and antigen thermal repair, 3% H_2_O_2_ solution was applied for 10 minutes to inactivate endogenous peroxidase, and 1% BSA was used for blocking. The primary antibodies of CAV-1, NF-κBp65, CD3, and F4/80 were incubated at 4 °C overnight, and corresponding secondary antibodies were added at room temperature and incubated for 2 h. Subsequently, the samples were incubated with DAB and hematoxylin staining solution. Finally, the samples were observed under a microscope and analyzed using ImageJ software (Bethesda, USA). The primary antibodies used are presented in Table S1.

### Cell culture and small-interfering RNA transfection

Bone marrow-derived macrophages (BMDMs) obtained from the mice were cultured in DMEM medium containing 30% L929-cell conditioned medium, 10% fetal bovine serum, and 1% penicillin/streptomycin, and the cells were placed in a humidified atmosphere at 37 °C with 5% CO_2_ for 7 days [29]. On day 4, the cells were treated with fresh medium, and on day 7 cells were seeded into new plates and incubated overnight. According to the instructions provided by the manufacturer, the cells were transfected with CAV-1-siRNA and scrambled-siRNA using a Lipofectamine 2000 system. After 24 h, the cells were co-incubated with 1 μg/mL of LPS, and were harvested after 24 h of LPS administration.

### RNA extraction and quantitative real-time PCR (qRT-PCR) analysis

Total RNA was extracted from mouse lung tissues using TRIzol reagent following the manufacturer’s instructions. Reverse transcription into cDNA was performed using a HiScript III RT SuperMix Kit (Vazyme, China). Real-time PCR analysis was conducted to detect the expression levels of TNF-α, IL-1β, IL-6, IL-18, CAV-1, NF-κBp65, and GAPDH using the SYBR Green qPCR Kit (Vazyme, Nanjing, China). The 2^−ΔΔCT^ method was used to quantify cDNA copy numbers. The mRNA levels were normalized to that of the reference gene, GAPDH. The primers used are presented in Table S2.

### Western blotting analysis

Lung tissue and cells were collected, and protein was extracted using a RIPA lysis buffer, and protein concentrations were measured using BCA (Beyotime, China). The proteins were loaded onto 10–12% SDS-PAGE and transferred onto PVDF membranes, which were blocked using 5% non-fat milk at room temperature for 1 h, and incubated with primary antibodies overnight at 4 °C. After washing the membrane with TBST three times, the membrane was incubated with the secondary antibody at room temperature for 1 h. Finally, a chemiluminescence reagent imaging system was used to detect the bands, and all the bands were measured using ImageJ software (Bethesda, USA). The primary antibodies used are shown in Table S1.

### Immunofluorescence analysis

The BMDMs were fixed using 4% paraformaldehyde, and permeated with 0.2% Triton X-100 for 10 minutes. Then, 5% bovine serum albumin was used for blocking for 1 h at room temperature. Subsequently, the cells were incubated with the primary antibodies, CAV-1 and NF-κBp65 (Table S1) overnight at 4°C, followed by incubation with the fluorescence secondary antibody for 1 h in the dark. The nuclei were stained using DAPI and the samples were sealed using the fluorescence quenching agent and observed under a confocal microscope (Leica, Germany).

### Transmission electron microscopic analysis

The samples were prepared according to standard protocols. In brief, the BMDMs were fixed using 2.5% glutaraldehyde solution at 4 °C for 2 h and 2% osmium tetroxide solution for 2 h. Then the sample was dehydrated using an acetone gradient, the cells were embedded, cut into ultrathin sections, the autophagosomes were stained and observed under a transmission electron microscopic (HITA-CHI, Japan).

### Statistical analyses

All the results are presented as mean ± SEM of at least four replicate experiments. Comparisons between groups were conducted using the Student’s *t* test or one-way ANOVA, and a *p* value of <0.05 was considered to indicate statistical significance.

## Results

### Identification of DEGs between ALI and normal controls using the GEO datasets

**Fig. 2:**
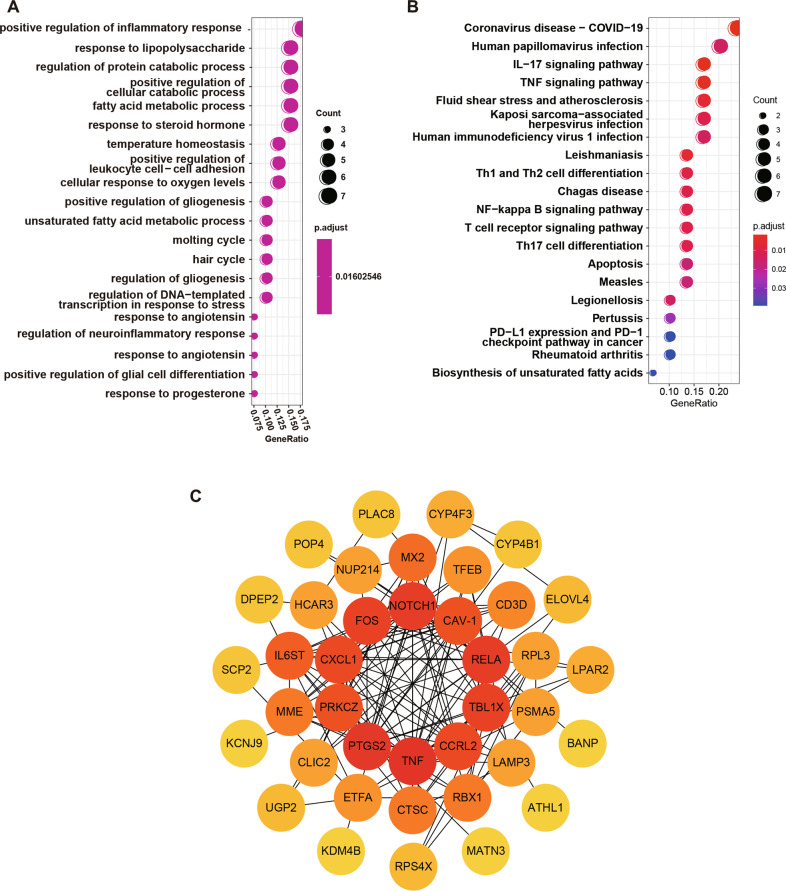
Functional enrichment and PPI network construction of DEGs. **A**, **B** Bubble plots show the results of GO functional enrichment and KEGG signaling pathway enrichment of DEGs. **C** The PPI network of the DEGs. Nodes represent proteins, edges represent interactions between proteins, and the intensity of the color indicates the degree of interaction of each protein. Proteins with more interaction were shown in the center of the network.

To identify the potential genes that are involved in ALI, five GEO datasets were analyzed and a total of 400 DEGs were identified. The subsequent RRA analysis found 40 robust DEGs, among which 20 genes were upregulated and the other 20 genes were downregulated (Table S3). In particular, CAV-1 and RELA expression levels were upregulated in the ALI groups (Fig. 1B, C). The functional enrichment analysis, included GO and KEGG pathways, and the most significantly enriched terms are shown in Fig. 2A, B. The 40 robust EDGs were primarily associated with the regulation of inflammatory response, response to lipopolysaccharide, protein catabolic process, and fatty acid metabolic process (Fig. 2A, Table S4). Meanwhile, the most related pathways included the IL-17 signaling pathway, TNF signaling pathway, and NF-κB signaling pathway (Fig. 2B, Table S5).

### CAV-1 and RELA were recognized as hub genes related to ALI

We constructed the PPI network of the 40 robust DEGs using the STRING website. The network contained 40 nodes and 136 edges, and was visualized using Cytoscape software (Fig. 2C). The top 10 proteins with the most interactions were: TNF, PTGS2, RELA, CAV-1, NOTCH1, TBL1X, FOS, CCRL2, CXCL1, and PRKCZ. Ultimately, we selected CAV-1 and RELA for further analysis, but the functions and molecular mechanisms of involvement of these two genes in ALI is unclear.

### Verification of the expression levels of CAV-1 and NF-κBp65 in vivo

**Fig. 3:**
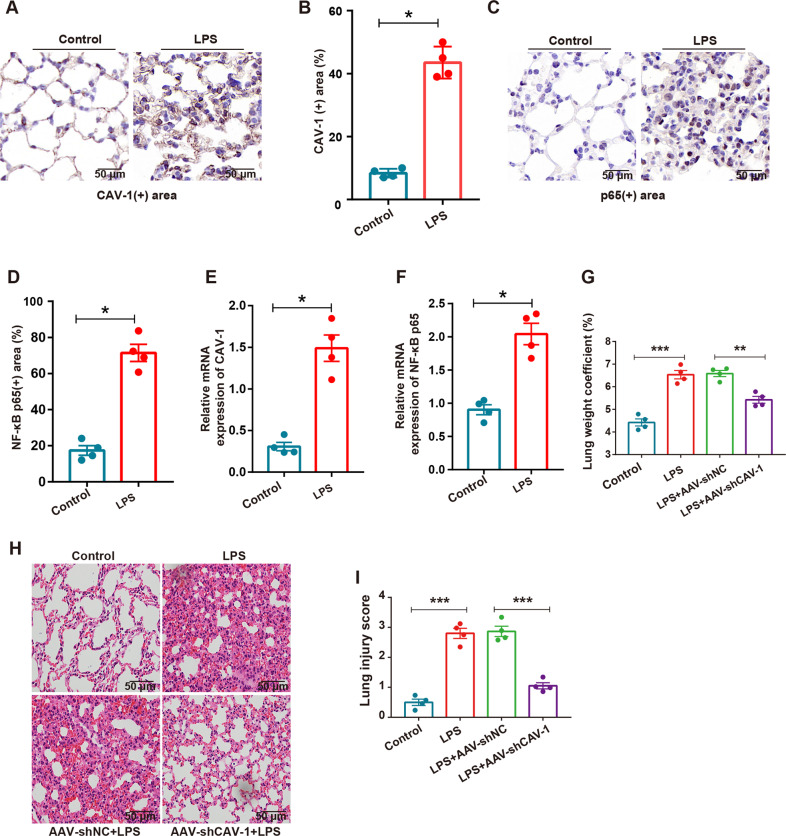
Expression of CAV-1 and NF-κB in mouse model of ALI. **A**–**D** The expression levels of CAV-1 and NF-κBp65 in lung tissues were analyzed by IHC. Scale bar = 50 μm. **E**, **F** qRT-PCR was performed to detect the levels of CAV-1 and NF-κBp65 in lung tissues. **G** The degree of pulmonary edema in each group was evaluated by lung weight coefficient. **H**, **I** H&E staining was used to detect the pathological changes in lung tissue and the score of morphological injury of lung tissue in each group. Scale bar = 50 μm. Results were represented as mean ± SEM (*n* = 4, *p < 0.05, **p < 0.01, ***p < 0.001).

To confirm the expression levels of CAV-1 and NF-κBp65 in ALI, we detected the expression levels of CAV-1 and NF-κBp65 in lung tissue. Immunohistochemical staining and quantitative analysis showed that LPS significantly increased the expression of CAV-1 and NF-κBp65 in lung tissues of mice compared with the control group (Fig. 3A–D). The expression levels of CAV-1 and NF-κBp65 were detected using qRT-PCR analysis, and a significant increase in CAV-1 and NF-κBp65 expression levels were observed following LPS treatment compared with the control group (Fig. 3E, F). These results are consistent with the results of our previous bioinformatics research, indicating that CAV-1 and NF-κBp65 may play important roles in the development of ALI.

### CAV-1 silencing ameliorated LPS-induced lung injury in mice

To explore the relationship between CAV-1 and the development of ALI, we treated ALI model mice with AAV carrying shCAV-1 to silence the expression of CAV-1. Compared with the control group, qRT-PCR and western blotting analysis confirmed that the expression of CAV-1 in the AAV-shCAV-1 group decreased by ~80% (Fig. S1A, B). The severity of lung injury was evaluated using the lung weight coefficient and H&E. The lung weight coefficient of LPS-induced ALI was significantly elevated, compared with the control. AAV-shCAV-1 injection could significantly decrease the lung weight coefficient after treatment with LPS (Fig. 3G). Accordingly, in the CAV-1 silenced mice treated with LPS, H&E staining showed that the infiltration of inflammatory cells decreased significantly, and the alveolar walls were significantly thinner (Fig. 3H, I). Overall, these results indicate that blocking CAV-1 could reduce ALI severity in the mice.

### CAV-1 silencing could promote autophagy in the LPS- induced lung injury model mice

**Fig. 4:**
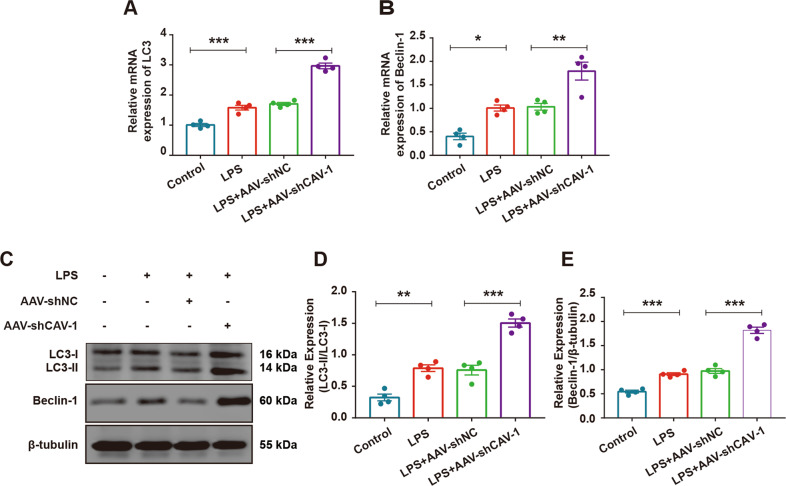
Knocking down of CAV-1 improved autophagy and ameliorated ALI. **A**, **B** qRT-PCR demonstrated the mRNA levels of LC3 and Beclin-1 in the lung tissues. **C** The protein levels of LC3II/I and Beclin-1 were examined by Western blotting in lung tissues. **D**, **E** Quantification of LC3 and Beclin-1 protein bands. Results were represented as mean ± SEM (n = 4, *p < 0.05, **p < 0.01, ***p < 0.001).

Autophagy plays an important role in the occurrence and development of ALI. To investigate the effect of CAV-1 on autophagy in the mice, we silenced CAV-1 in the mice using AAV-shCAV-1 to observe its influence on autophagy-related proteins. The results showed that both the mRNA and the protein levels of LC3II/I, Beclin-1, and Atg5 increased and p62 decreased after LPS stimulation. Meanwhile, the expression levels of LC3II/I, Beclin-1, and Atg5 were further upregulated and p62 was significantly downregulated after the administration of AAV-shCAV-1 (Fig. 4A–E, Fig. S2A–F), suggesting that CAV-1 silencing could promote autophagy and ameliorate LPS-induced lung injure in mice.

### CAV-1 silencing inhibited the pulmonary inflammatory response

**Fig. 5:**
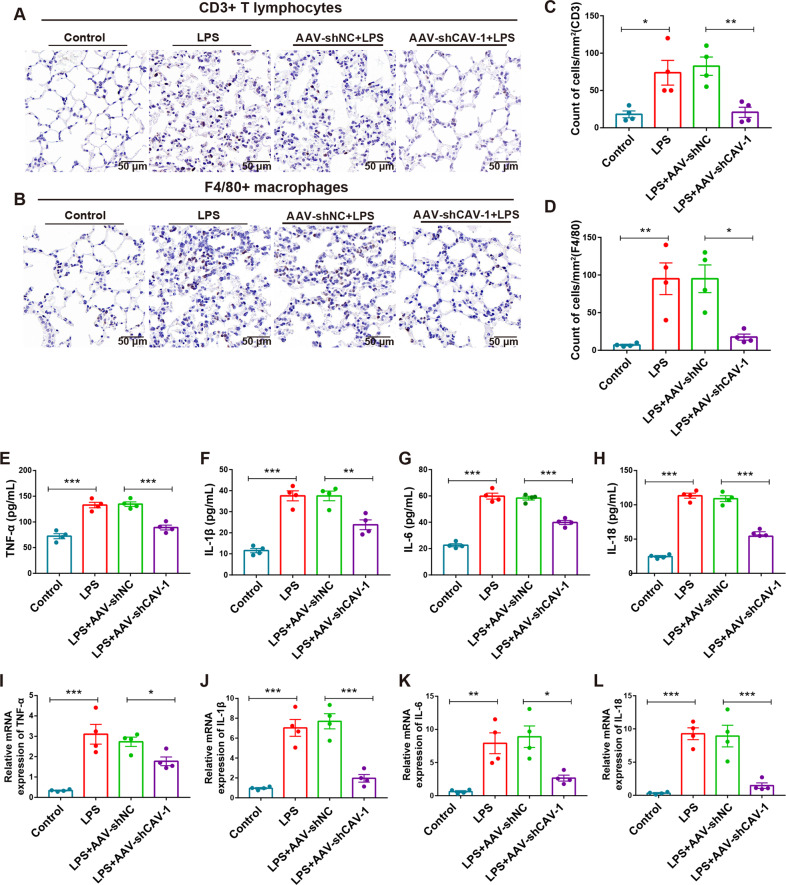
Knockdown of CAV-1 could reduce the infiltration of CD3^+^ T cells and F4/80^+^ macrophages and inhibit proinflammatory cytokines. **A**, **C** The expression of CD3 protein in T cells was detected by immunohistochemistry and the quantification of CD3^+^ T cells. **B**, **D** The protein levels of F4/80 was determined by IHC and quantification of F4/80^+^ cells. Scale bar = 50 μm. **E**–**H** The secretion of TNF-α, IL-1β, IL-6, and IL-18 in serum were determined by ELISA. **I**–**L** qRT-PCR analysis of TNF-α, IL-1β, IL-6, and IL-18 in lung tissues. Results were represented as mean ± SEM (*n* = 4, *p < 0.05, **p < 0.01, ***p < 0.001).

Subsequently, to explore the immune mechanism of CAV-1 in LPS-induced ALI, we evaluated whether CAV-1 silencing could regulate pulmonary inflammation. According to our immunohistochemistry results of the mice lung tissues obtained from each group, LPS treatment increased the infiltration of CD3^+^ T lymphocytes compared with the control, while AAV-shCAV-1 administration significantly reversed the accumulation of CD3^+^ T cells (Fig. 5A, C). Similar results were observed with the amount of F4/80^+^ macrophages (Fig. 5B, D), indicating that CAV-1 knockdown prevented immune cell infiltration. Meanwhile, we evaluated the secretion of proinflammatory cytokines in ALI. The levels of IL-1β, IL-6, IL-18, and TNF-α were upregulated in the LPS-treated mice compared with control mice, while AAV-shCAV-1 injection could significantly decrease these expression levels (Fig. 5E–H). Similar results were observed with the mRNA expression levels of the proinflammatory cytokines in lung tissues (Fig. 5I–L). These results indicate that the silencing CAV-1 using AAV-shCAV-1 could suppress the excessive immune response in LPS-induced ALI.

### CAV-1 knockdown upregulated the level of autophagy in the BMDMs

**Fig. 6:**
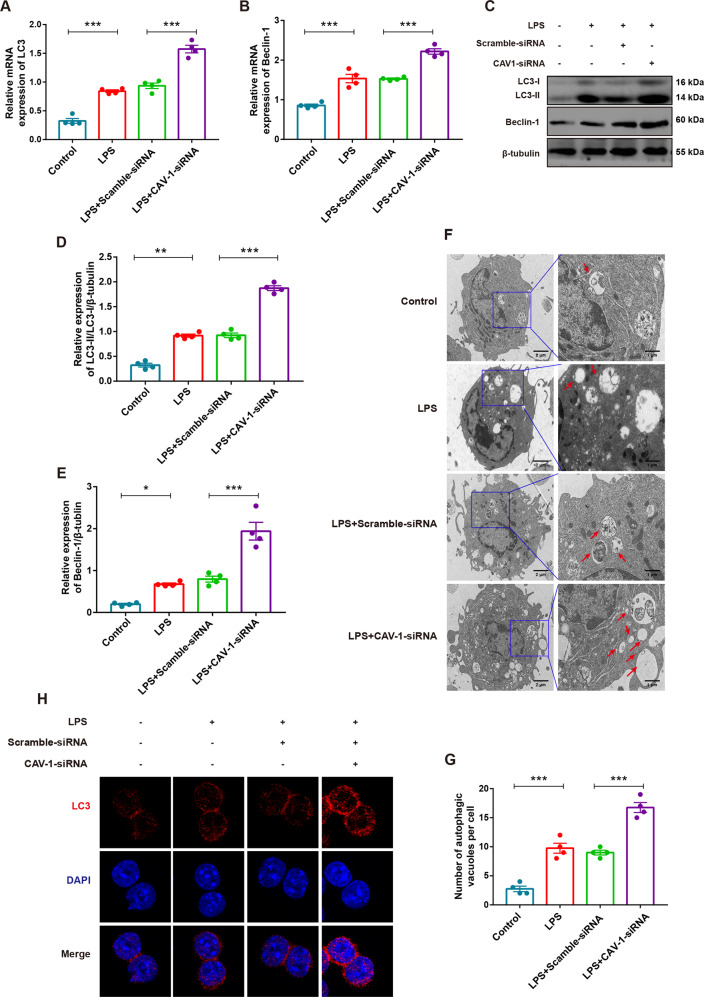
Knockdown of CAV-1 activated autophagy in BMDMs. **A**, **B** The mRNA levels of LC3 and Beclin-1 by qRT-PCR in BMDMs. **C** The protein expression of LC3II/I and Beclin-1 were assessed by western blotting in BMDMs. **D**, **E** Quantification of LC3 and beclin-1 expression. **F** The formation of autophagosomes in different groups of BMDMs was observed by TEM. Red arrows indicate autophagosomes. **G** Quantitative analysis of BMDMs autophagosomes number in each group. **H** Immunofluorescence analysis of LC3 (red) expression in BMDMs (magnification ×63). Results were represented as mean ± SEM (*n* = 4, *p < 0.05, **p < 0.01, ***p < 0.001).

To investigate the role of CAV-1 in BMDMs, two pairs of CAV-1-siRNA were transfected into BMDMs to interfere with the expression of CAV-1. As shown in Fig. S3A, B, qRT-PCR analysis and western blotting showed that the expression of CAV-1 decreased by ~85% in the CAV-1-siRNA group, compared with the control groups. CAV-1 and autophagy play vital regulatory roles in ALI. To more closely elucidate the function of CAV-1 on autophagy, CAV-1 was knocked down in the LPS-treated cells using CAV-1-siRNA. The mRNA and protein levels of LC3II/I and Beclin-1 were upregulated, compared with the control groups. The knockdown of CAV-1 could further increase these mRNA and protein expression levels after LPS treatment (Fig. 6A–E). Furthermore, cells in the LPS groups exhibited a larger number of autophagosomes in BMDMs, compared with the control groups. Autophagosome formation increased significantly in the CAV-1 knockdown BMDMs after LPS treatment (Fig. 6F, G). Meanwhile, immunofluorescence assays showed that autophagy was activated by increasing the formation of LC3 puncta after stimulation by LPS, compared with the controls. CAV-1 knockdown could markedly increase the level of LC3 after treatment with LPS (Fig. 6H). These results suggest that the knockdown of CAV-1 promotes the activation of autophagy in BMDMs.

### CAV-1 knockdown inhibited the AKT/mTOR and promoted AMPK signaling pathways

**Fig. 7:**
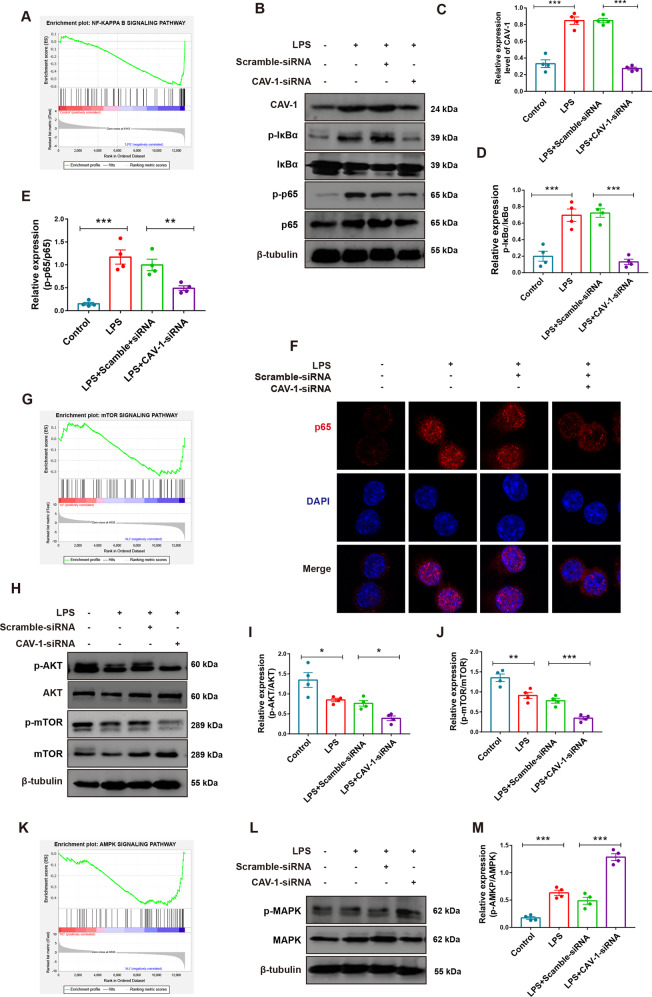
Knockdown of CAV-1 inhibited autophagy-related signaling pathways in BMDMs. **A** GSEA analysis of NF-κB signaling pathway in ALI samples. **B** Western blotting analysis of CAV-1, p-IκBα, IκBα, p-NF-κBp65, and NF-κBp65 protein levels in BMDMs. **C**–**E** Quantification of these protein bands. **F** Confocal laser immunofluorescence showed NF-κBp65 nuclear translocation in BMDMs from different groups (magnification ×63). **G** GSEA analysis of mTOR signaling pathway in ALI sample. **H**, **L** The expression of p-AKT, AKT, p-mTOR, mTOR, p-AMPK, and AMPK were detected by western blotting in BMDMs**. I**, **J**, **M** Quantification of these protein bands. **K** GSEA analysis of AMPK signaling pathway in ALI samples. Results were represented as mean ± SEM (*n* = 4, *p < 0.05, **p < 0.01, ***p < 0.001).

Autophagy-related pathways AKT/mTOR and AMPK play important roles in many diseases. To elucidate the precise mechanism by which CAV-1 promotes ALI, we determined whether CAV-1 expression was associated with the NF-κB, AKT/mTOR, and AMPK signal pathways, which are crucial regulators of autophagy. Gene set enrichment analysis showed that NF-κB, mTOR, and AMPK signaling pathway was significantly enriched in ALI group (Fig. 7A, G, K). Western blotting revealed that CAV-1, IκBα, and NF-κBp65 were significantly activated after LPS administration, compared with the control, and this effect was significantly attenuated by CAV-1 knockdown (Fig. 7B–E). Meanwhile, we analyzed the nuclear translocation of NF-κBp65 using immunofluorescence staining, and CAV-1 knockdown significantly reduced LPS-induced nuclear translocation of NF-κBp65 (Fig. 7F). Furthermore, AKT and mTOR phosphorylation levels in the LPS exposed DMBMs decreased, and these phosphorylation levels were more significantly attenuated after CAV-1 knockdown (Fig. 7H–J). Subsequently, the phosphorylation level of AMPK increased significantly after LPS administration, while transfection with CAV-1-siRNA effectively reduced the level of AMPK phosphorylation (Fig. 7L, M). Taken together, we demonstrated that CAV-1 knockdown could increase autophagy in part by inhibiting the NF-κB and AKT/mTOR pathway and promoting the AMPK pathway.

## Discussion

**Fig. 8:**
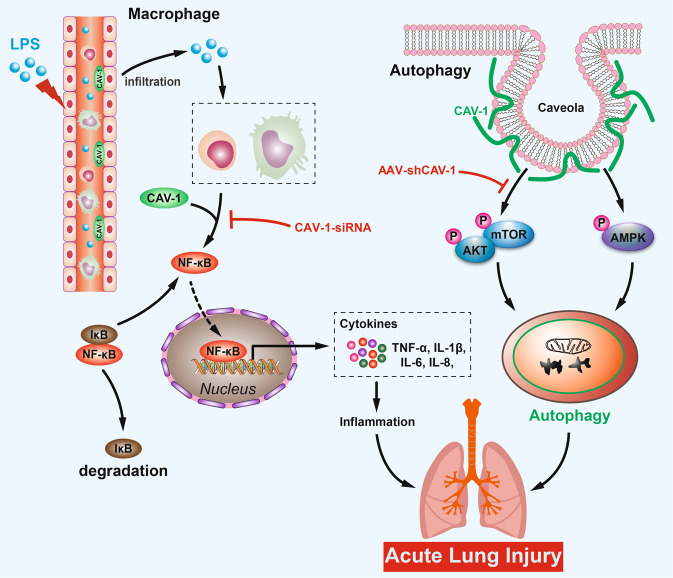
Molecular mechanism of CAV-1-mediated LPS-induced ALI development. CAV-1 expression is upregulated in LPS-induced ALI, which recruits CD3^+^ T lymphocytes and F4/80^+^ macrophages to lung tissue, thereby triggering inflammatory response. Meanwhile, upregulated CAV-1 could promote AKT/mTOR and inhibit AMPK to downregulate autophagy, resulting in ALI. Conversely, knockdown of CAV-1 ameliorates ALI.

In this study, we found that activation of the CAV-1/NF-κB axis was associated with LPS-induced inflammatory response and autophagy in ALI. Inhibition of the CAV-1/NF-κB axis through CAV-1 knockdown could markedly reduce the severity of LPS-induced ALI by promoting the autophagy-related pathway, AMPK, and by inhibiting the activation of the AKT/mTOR pathway and the inflammatory response caused by the infiltration of CD4/80^+^ macrophages and CD3^+^ T lymphocytes. Taken together, these results indicate that the CAV-1/NF-κB axis could be a potential therapeutic target for ALI (Fig. 8).

We first used bioinformatics analysis to identify CAV-1 and NF-κB as key genes among the DEGs that were significantly upregulated in ALI. CAV-1 is a protein located on cell membranes that can regulate cell signaling pathways through certain intracellular signal transduction molecules [30]. Each signaling pathway converges in NF-κB, which is a key pathway of the inflammatory response that contributes to the occurrence of ALI [31, 32]. He et al. [33] found that the CAV-1/NF-κB pathway could increase macrophage infiltration into lung tissue and promote the occurrence of ALI, while CAV-1 deficiency aggravated ALI. The CAV-1/NF-κB axis is also involved in various pathological processes, such as tumorigenesis [34], inflammatory response [32], aerobic glycolysis [35], autophagy [36], and apoptosis, and may be a promising therapeutic target for ALI treatment. At present, few studies have reported that CAV-1 is associated with ALI, and its precise mechanism is not fully understood [37]. Our in vivo and in vitro experiments identified that the hyperactivation of the CAV-1/NF-κB axis was associated with ALI, which was consistent with the results of the bioinformatics analysis. Subsequently, CAV-1 knockdown was used to explore its molecular mechanism in ALI through autophagy and inflammatory response.

Immune cell infiltration and inflammatory responses play an important role in ALI [38, 39]. In sepsis-induced ALI, immune cells migrate through the vessels into the alveoli, causing pulmonary endothelial cell damage that leads to ALI [20]. Leukocytes and macrophages play a crucial part in ALI development [40]. Meanwhile, the infiltration of innate immune cells contributes to the early-stage secretion of cytokines, including TNF-α, IL-1β, IL-6, IL-10, and IL-18, which further induced an excessive immune response in the alveoli to aggravate ALI [41–43]. CD3^+^ T cells are also involved in ALI through both direct tissue damage and indirect injury by producing inflammatory factors [44]. CAV-1 and NF-κB have been reported to participate in the development of ALI [22]. Garrean et al. [22] reported that CAV-1 could control the activation of NF-κB, which promotes the inflammation response in ALI. Xu L et al. [23] showed that Dex administration promoted the expression of CAV-1 and suppressed the activation of NF-κB, thus reducing LPS-induced pulmonary injury. However, the precise mechanism by which the CAV-1/NF-κB axis is involved in ALI remains unclear. In our study, we showed that the infiltration of CD3^+^ T cells and F4/80^+^ macrophages increased after LPS administration, which is consistent with the results of previous studies. Importantly, CAV-1 knockdown by AAV-shCAV-1 in mice suppressed elevated recruitment of T cells and macrophages. Meanwhile, LPS administration promoted the secretion of TNF-α, IL-1β, IL-6, and IL-18, but AAV-shCAV-1 decreased the expression levels of these cytokines as well as their mRNA level expression. Interestingly, we verified that the knockdown of CAV-1 reduced the activation and nuclear translocation of NF-κBp65. Considering that NF-κB is a crucial mediator of inflammatory factor transcription, we suggest that the CAV-1/NF-κB axis may regulate immune infiltration and cytokine secretion.

Autophagy, a process by which proteins and damaged organelles are degraded, plays a critical role in maintaining the homeostasis of the intracellular environment and cell survival [45, 46]. Autophagy is reportedly relevant in multiple processes, including cell renovation, inflammation, and pathogen defense [47], and plays a crucial role in various diseases, such as neurodegenerative disorders and cancers [48]. In the process of autophagy formation, the cytoplasmic LC3-I was transformed into LC3II, and the expression of autophagy markers Atg5 and Beclin-1 increased [49]. Meanwhile, p62 could be degraded by proteolytic enzymes in autophagolysosomes, and the expression of p62 is negatively correlated with autophagy activity [50, 51]. In recent years, autophagy has been shown to play a crucial role in lung diseases [52]. Selective autophagy is associated with NF-κB activation and induces inflammation, which plays a part in ALI [53]. In addition, autophagy could directly participate in ALI by regulating NALP3-dependent inflammation [54]. Chen et al. [55] demonstrated that CAV-1 could bind to ATG12-ATG5, and that the knockdown of CAV-1 led to the dissociation of the complex and induced autophagy in lung epithelial cells. However, the mechanism by which CAV-1 and autophagy are involved in ALI remains to be explored. In our study, in vivo, and in vitro experiments showed that CAV-1 knockdown promoted autophagy-related proteins LC3II/I, Beclin-1, Atg5 and downregulation of p62 expression in ALI. To further investigate the mechanism involved, we examined the activation of autophagy-related pathways. Our results indicated that CAV-1 downregulation suppressed the AKT/mTOR pathway and activated autophagy, while the AMPK pathway was promoted.

In conclusion, this study confirmed the role of CAV-1/NF-κB in ALI, and demonstrated the mechanism by which CAV-1 knockdown upregulated autophagy to attenuate LPS-induced ALI. Nevertheless, the relationship between CAV-1 and autophagy in ALI is still not clear. Therefore, future research should focus on clarifying the detailed mechanism to support its clinical application.

## Conclusions

Taken together, this study showed that activation of the CAV-1/NF-κB axis in LPS-induced ALI was associated with a decrease in autophagy and an increase in inflammation in the lung tissue. CAV-1 knockdown could block the CAV-1/NF-κB axis to significantly suppress ALI by inhibiting the autophagy-related signaling pathway, AKT/mTOR, and promoting AMPK activation, as well as inhibiting the inflammatory response caused by the infiltration of CD3^+^ and F4/80^+^ cells. However, further functional experiments need to be performed to gain a deeper understanding of the underlying mechanisms and support the blockade of the CAV-1/NF-κB axis, which may provide broad prospects for the future clinical treatment of ALI.

## Supplementary information

Supplementary figure legends

Supplementary Figure1

Supplementary Figure2

Supplementary Figure3

Supplementary_Table1

Supplementary_Table2

Supplementary_Table3

Supplementary_Table4

Supplementary_Table5

Supplementary Material-western blots

checklist

## Data Availability

All data required for the conclusion of the manuscript can be found in the article. Additional raw data related to this study are available on request from corresponding authors.
