# Supplementary material for: Caveolin-1 identified as a key mediator of acute lung injury using bioinformatics and functional research
Source: Cell Death Dis. 2022 Aug 6;13(8):686. doi: 10.1038/s41419-022-05134-8 (PMC9357074; doi:10.1038/s41419-022-05134-8)
Supplement: Supplementary file 1 — Supplementary figure legends [file 41419_2022_5134_MOESM1_ESM.docx]

**Supplementary figure legends**

**Fig. S1** **Knockdown of CAV-1 in mice.** **A-B** qRT-PCR and Western blot analysis were used to detect the knockdown efficiency of CAV-1 mRNA and protein levels in AAV-shNC and AAV-shCAV-1. Results were represented as mean ± SEM (n = 4, ****p* < 0.001, compared with control).

**Fig. S2 Knocking down of CAV-1 improved autophagy and ameliorated ALI. A-B** qRT-PCR analysis demonstrated the mRNA levels of Atg5 and p62 in the lung tissues. **C, E** The protein levels of Atg5 and p62 were examined by Western blotting in lung tissues. **D, F** Quantification of Atg5 and p62 protein bands. Results were represented as mean ± SEM (n = 4, **p* < 0.05, ***p* < 0.01, ****p* < 0.001)

**Fig. S3** **Knockdown of CAV-1 in BMDMs.** **A-B** The mRNA and protein levels of CAV-1 were detected by qRT-PCR analysis and Western blotting in Scramble-siRNA and CAV-1-siRNA. Results were represented as mean ± SEM (n = 4, ****p* < 0.001, compared with control).
