# Supplementary figures and images for: Caveolin-1 identified as a key mediator of acute lung injury using bioinformatics and functional research
Source: Cell Death Dis. 2022 Aug 6;13(8):686. doi: 10.1038/s41419-022-05134-8 (PMC9357074; doi:10.1038/s41419-022-05134-8)

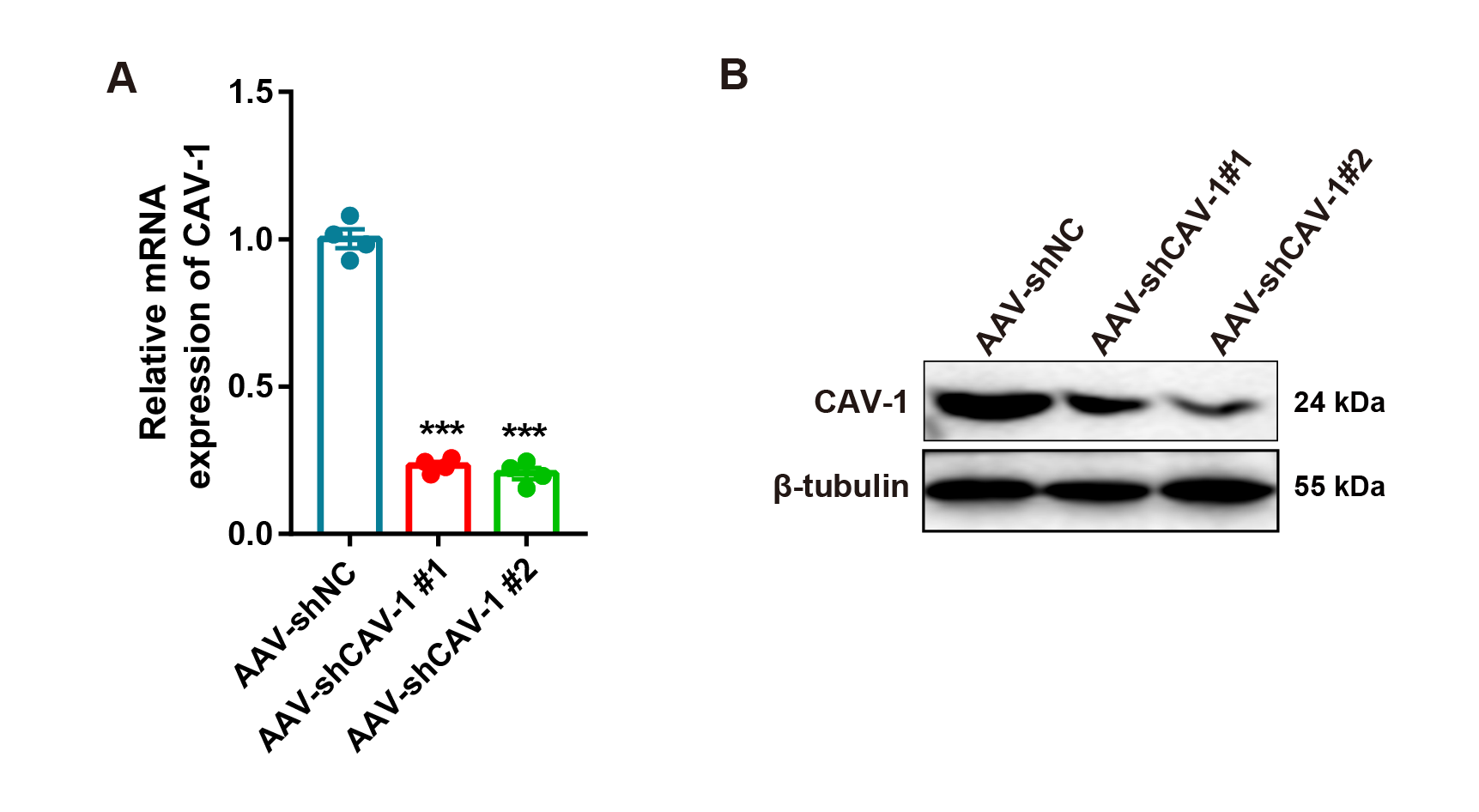

Supplement: Supplementary file 2 — Supplementary Figure1 [file 41419_2022_5134_MOESM2_ESM.tif]

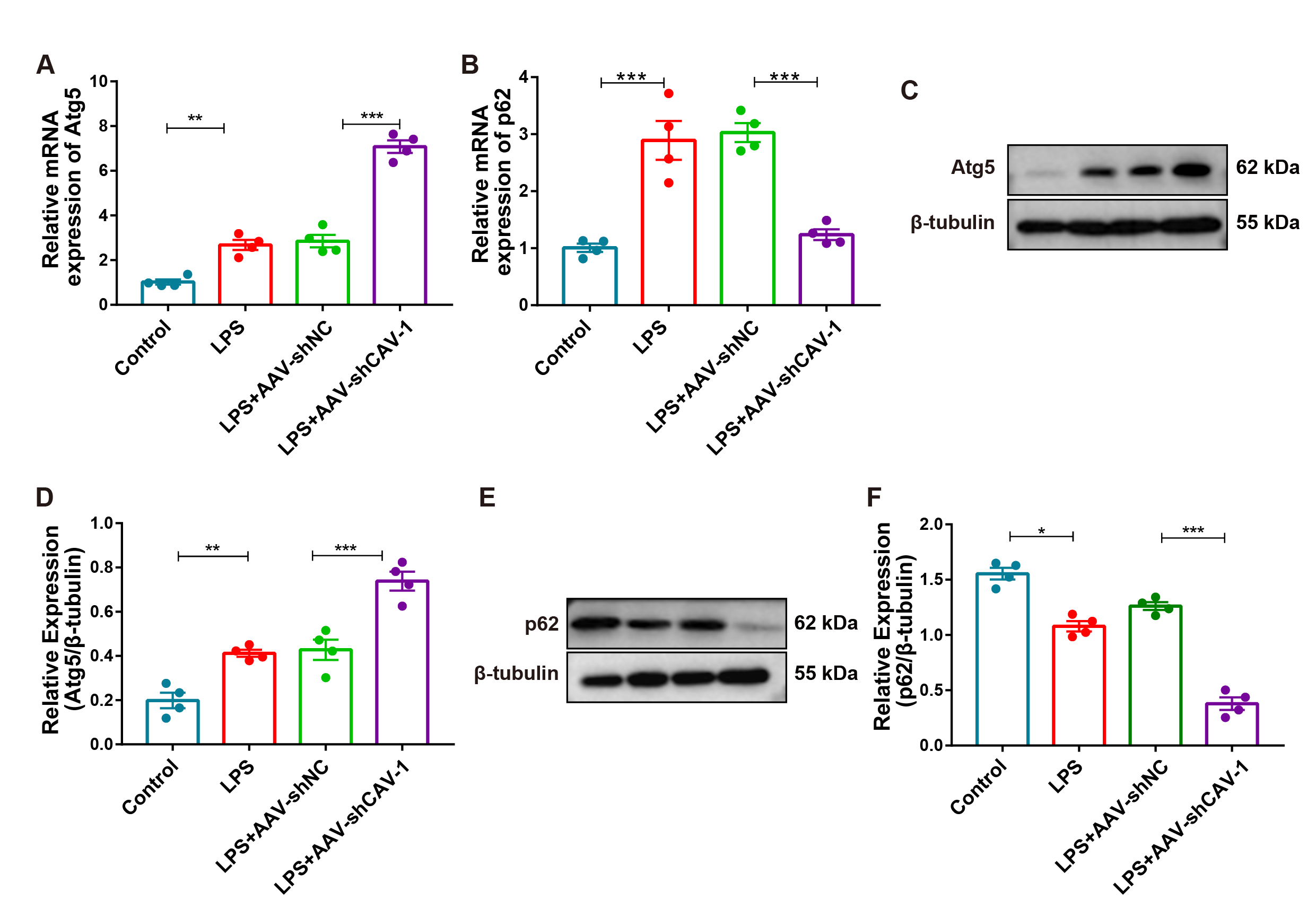

Supplement: Supplementary file 3 — Supplementary Figure2 [file 41419_2022_5134_MOESM3_ESM.tif]

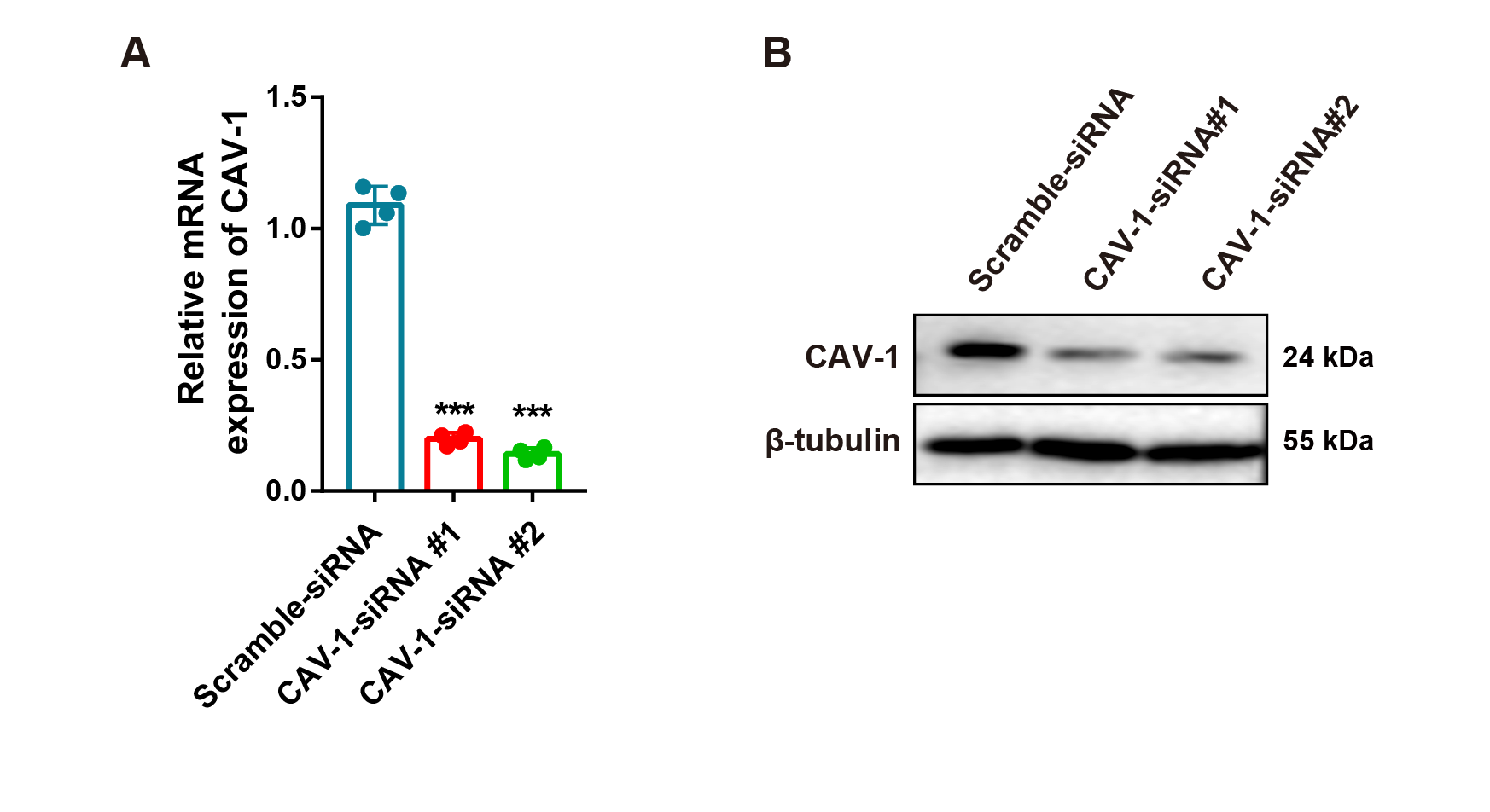

Supplement: Supplementary file 4 — Supplementary Figure3 [file 41419_2022_5134_MOESM4_ESM.tif]
