# Supplementary material for: Caveolin-1 identified as a key mediator of acute lung injury using bioinformatics and functional research
Source: Cell Death Dis. 2022 Aug 6;13(8):686. doi: 10.1038/s41419-022-05134-8 (PMC9357074; doi:10.1038/s41419-022-05134-8)
Supplement: Supplementary file 5 — Supplementary_Table1 [file 41419_2022_5134_MOESM5_ESM.docx]

**Table S1**. Primary antibodies used in the study.

| Antibody | MV (KDa) | Sources (Catalogue #) | Dilution |
| --- | --- | --- | --- |
| Caveolin-1 | 24 | Cell Signaling Technology (3267) (USA) | WB: 1:1000; IHC: 1:500 |
| IκBα | 36 | Cell Signaling Technology (4812) (USA) | WB: 1:1000 |
| p-IκBα | 36 | Cell Signaling Technology (2859) (USA) | WB: 1:1000 |
| NF-κBp65 | 65 | Cell Signaling Technology (8242) (USA) | WB: 1:1000; IHC: 1:500; IF:500 |
| p-NF-κBp65 | 65 | Cell Signaling Technology (3033) (USA) | WB: 1:1000 |
| LC3B | 14,16 | Proteintech(18725-1-AP) (China) | WB: 1:1000; IF: 1:200 |
| Beclin-1 | 60 | Proteintech (11306-1-AP) (China) | WB: 1:2000 |
| CD3 | - | Abcam(ab16669) (USA) | IHC: 1:100 |
| F4/80 | - | Cell Signaling Technology (70076) (USA) | IHC: 1:200 |
| AKT | 60 | Proteintech(10176-2-AP) (China) | WB: 1:2000 |
| p-AKT | 60 | Cell Signaling Technology (4060) (USA) | WB: 1:2000 |
| mTOR | 289 | Proteintech (10176-2-AP) (China) | WB: 1:1000 |
| p-mTOR | 289 | Cell Signaling Technology (5536) (USA) | WB: 1:1000 |
| AMPKα | 62 | Cell Signaling Technology (5831) (USA) | WB: 1:1000 |
| p-AMPKα | 62 | Cell Signaling Technology (2535) (USA) | WB: 1:1000 |
| Atg5 | 62 | Cell Signaling Technology (9980) (USA) | WB: 1:1000 |
| p62 | 62 | Cell Signaling Technology (8025) (USA) | WB: 1:1000 |
| β-tubulin | 55 | Servicebio (GB11017) (China) | WB: 1:2000 |
