# Supplementary material for: Caveolin-1 identified as a key mediator of acute lung injury using bioinformatics and functional research
Source: Cell Death Dis. 2022 Aug 6;13(8):686. doi: 10.1038/s41419-022-05134-8 (PMC9357074; doi:10.1038/s41419-022-05134-8)
Supplement: Supplementary file 6 — Supplementary_Table2 [file 41419_2022_5134_MOESM6_ESM.docx]

**Table S2**. Primer sets used for qRT-PCR analysis.

| Gene | Forward Primer (5' to 3') | Reverse Primer (5' to 3') |
| --- | --- | --- |
| Caveolin‑1 | TGACTGAGAAGCAAGTGTATGA | AGGAGAGAATGGCAAAGTAAAT |
| NF-κBp65 | TGATGTGCATCGGCAAGTGG | CAGAAGTTGAGTTTCGGGTAGGC |
| LC3 | AGCTTCGCCGACCGCTGTAA | ATCCGTCTTCATCCTTCTCCTGTTC |
| Beclin-1 | CCAATAAGATGGGTCTGAAGTT | ATCCGTCTTCATCCTTCTCCTGTTC |
| TNF-α | AGTTCCCAAATGGCCTCCCTC | GGGCTACAGGCTTGTCACTCG |
| IL-1β | TCAAATCTCGCAGCAGCACAT | TCATCTCGGAGCCTGTAGTGC |
| IL-6 | ACAACCACGGCCTTCCCTAC | CTTTTCTCATTTCCACGATTTCCC |
| IL-18 | TTTATTGACAACACGCTTTAC | CTTTTGTCAACGAAGAGAACT |
| GAPDH | CTCATGCGCTGTGTGGAA | GAAAATGGGAAACTGGCT |
