# Supplementary material for: Caveolin-1 identified as a key mediator of acute lung injury using bioinformatics and functional research
Source: Cell Death Dis. 2022 Aug 6;13(8):686. doi: 10.1038/s41419-022-05134-8 (PMC9357074; doi:10.1038/s41419-022-05134-8)

**Fig.4C**


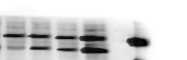


**LC3**


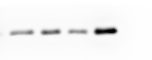


**β-tubulin**

**Beclin-1**


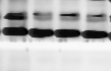


**Fig.6C**


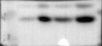


**LC3**


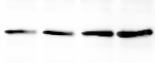


**Beclin-1**

**β-tubulin**


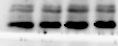


**Fig.7B**

**CAV-1**


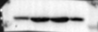


**p-IκBα**


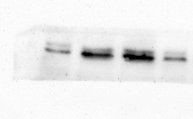


**
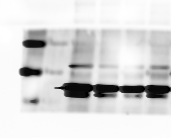
**

**IκBα**


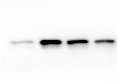


**p-p65**


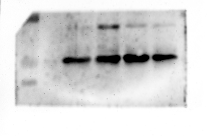


**p65**

**β-tubulin**


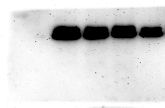


**Fig.7H**

**p-AKT**


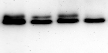


**AKT**


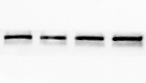


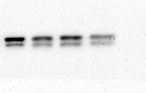


**p-mTOR**


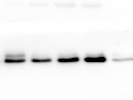


**mTOR**


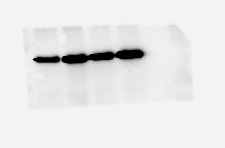


**β-tubulin**

**Fig.7L**

**p-AMPK**


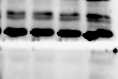


**AMPK**


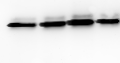


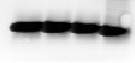


**β-tubulin**

**Supplementary figure**

**Fig. S1B**


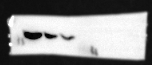


**β-tubulin**

**CAV-1**


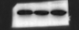


**Fig. S2C**

**Atg5**


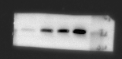


**β-tubulin**


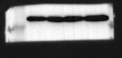


**Fig. S2E**

**p62**


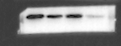


**β-tubulin**


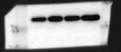


**Fig. S3B**

**CAV-1**


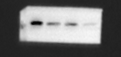


**β-tubulin**


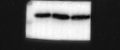

Supplement: Supplementary file 10 — Supplementary Material-western blots [file 41419_2022_5134_MOESM10_ESM.docx]
